# Effects of Acceptance and Commitment Therapy and Repetitive Transcranial Magnetic Stimulation on Obsessive–Compulsive Disorder

**DOI:** 10.3389/fpsyt.2021.720518

**Published:** 2022-01-12

**Authors:** Jingzhi Zou, Siliang Wu, Xin Yuan, Zhizhong Hu, Jun Tang, Maorong Hu

**Affiliations:** ^1^Department of Psychosomatic Medicine, The First Affiliated Hospital of Nanchang University, Nanchang, China; ^2^Department of Psychiatry, Xiamen Xianyue Hospital, Xiamen, China

**Keywords:** obsessive-compulsive disorder, acceptance and commitment therapy, repetitive transcranial magnetic stimulation, sertraline hydrochloride, psychological flexibility

## Abstract

**Objective:** This study aims to explore the difference of clinical efficacy and psychological flexibility of sertraline hydrochloride combined with acceptance and commitment therapy (ACT) or repeated transcranial magnetic stimulation (rTMS) in patients with obsessive–compulsive disorder (OCD).

**Materials and Methods:** Sixty-three inpatients diagnosed with OCD were randomly divided into ACT group (*N* = 32) and rTMS group (*N* = 31), both of which were combined with sertraline hydrochloride. The following assessments were completed by the Hamilton Depression Scale (HAMD), Hamilton Anxiety Scale (HAMA), Yale–Brown Obsessive Compulsive Scale (Y-BOCS), Symptom Checklist 90 (SCL-90), Acceptance and Action Questionnaire (AAQ-II), and Cognitive Fusion Questionnaire (CFQ) during pretreatment, 4 weeks posttreatment, and 8-week follow-up.

**Results:** After treatment: (1) the SCL-90 score of two groups significantly decreased from pretreatment to 8-week follow-up (*P* < 0.01 and *P* < 0.001); (2) The HAMA, HAMD, and Y-BOCS scores of the two groups significantly decreased from pretreatment to 8-week follow-up (*P* < 0.001 and *P* < 0.05); (3) No statistically significant difference of the SCL-90, HAMA, HAMD and Y-BOCS between two groups; (4) The AAQ-II and CFQ scores of the ACT group significantly decreased from 4 weeks posttreatment to 8-week follow-up (*P* < 0.01). However, no statistically significant difference was observed in the rTMS group (*P* > 0.05).

**Conclusions:** Overall, our study suggested that sertraline hydrochloride combined with ACT or rTMS can improve the obsessive–compulsive symptoms, anxiety, and depression and has equivalent efficacy. Moreover, ACT can more effectively and durably improve the psychological flexibility of patients compared with rTMS.

## Background

Obsessive–compulsive disorder (OCD) is a severe mental disorder that is characterized by repetitive obsessive–compulsive behaviors (compulsions) and intrusive thoughts (obsessions). Epidemiological studies have found that the prevalence of OCD is 2–3%. Severely affected patients may spend a substantial amount of time daily on compulsions and obsessions, which seriously influence people's social functions and quality of life (1). In regard to the pathogenesis of OCD, a large number of relevant studies have been carried out worldwide; however, it is still obscured.

Many neurotransmitter systems, such as 5-hydroxytryptamine (5-HT), dopamine systems, and their downstream signaling pathways, are related to the pathophysiological mechanism of OCD (2). 5-hydroxytryptamine may regulate some upstream executive functions, such as cognitive flexibility. Some animal experimental studies (3, 4) have shown that 5-HT can regulate the reversal learning function in the orbitofrontal cortex, and the release of which is influenced by dopaminergic stimulation through D2-like receptors in the striatum. Depletion of 5-HT can maintain reversal learning, similar to a form of the compulsion, which is consistent with the reversal deficit of patients (3, 5). The pharmacological therapy of OCD is mainly based on the modulation of neurotransmitters, especially the 5-HT and dopamine systems. Selective serotonin reuptake inhibitors (SSRIs) are considered to be the basis of drug therapy. More than 20 trials (6) have explicitly defined the efficacy of single SSRI in the treatment of OCD. SSRIs are the first-line choice for OCD drugs by considering efficacy and side effects. The use of high-dose SSRIs, which exceeds the usual dose range recommended by the supplier, will have a better effect in the treatment of OCD. A meta-analysis (7) clearly revealed the benefits of the treatment of higher doses. However, the molecular mechanism of the SSRIs is still unclear. Available evidence supports the idea that SSRIs may play a role in OCD by indirectly improving reward and punishment learning performance, thereby enhancing patients' ability to cope with compulsions (8). Patients suffering from OCD must insist on long-term medication therapy because sudden withdrawal is closely related to the high recurrence rate of OCD symptoms (9). In addition, using a single SSRI to alleviate OCD symptoms takes a longer time to work compared with the treatment of depression. A large number of experimental studies suggest that this process takes approximately 8–12 weeks (10). A more significant problem is that 40–50% of patients show extremely poor responses to the first-line drug therapy, and even a larger proportion of patients' symptoms cannot be completely relieved (11).

Although the neurobiological mechanism and etiology of OCD are still largely unknown, its dominant pathogenesis model focuses on defects in cognitive and behavioral inhibition and dysfunctions, both of which are related to the cortex–striatum–thalamic circuit (1). Neurophysiological and neuroimaging studies have shown that the motor and pre-motor areas of patients suffering from OCD are hyperactive (12, 13), and the result is consistent with this model. In particular, the supplementary motor area (SMA) is closely relevant to functional areas involved in cognitive and motor functions (14, 15) and plays a role in response regulation (14), which indicates that the functional area of OCD patients are overactive in performing response inhibition (16). Transcranial magnetic stimulation (TMS) is an emerging non-invasive neuromodulation technique, which is suggested to treat major depression disorder and OCD, moreover, TMS has a lower cost and security risk compared with deep brain stimulation (17). Repetitive TMS (rTMS) stimulates the coils close to the scalp to generate repetitive, short-lived, powerful magnetic pulses, which depolarize neurons. Then, the depolarization of neurons triggers a series of neurophysiological changes, indirectly giving rise to induced currents in the brain. High frequency (>5 Hz) can enhance the excitability of the cortex; however, low frequency (≤1 Hz) can inhibit the excitability of the cortex (18). Studies have found that not all patients respond to SSRIs, evidence showed that performing rTMS treatment on some patients with drug resistance will improve patients' obsessive–compulsive symptoms (OCS) (19). Since 1997, rTMS therapy has been used to treat refractory patients, and the therapeutic effect also slightly varies according to the frequency and location of stimulation (20). Relevant studies on rTMS therapy with low frequency and targeting SMA area suggest the partial effect on OCS (21).

Exposure and response prevention (ERP) gradually exposes patients to anxiety-inducing stimuli and makes patients refrain from ritual or avoidance behaviors. And cognitive behavior therapy (CBT) added cognitive restructuring to exposure exercises (22). CBT combined with antidepressants (SSRIs) are the first line treatment for children and adolescents with OCD, however, this therapy is not effective for all patients (23). Acceptance and commitment therapy (ACT) is a representative therapy in the third generation of behavioral therapy and focuses on reducing experiential avoidance and increasing psychological flexibility (24). Experiential avoidance refers to the avoidance of unpleasant events, such as thoughts, emotions, and memories (25). Numerous studies have shown that experiential avoidance plays an important role in the development and maintenance of OCD. From this perspective, patients are committed to controlling or reducing their obsessions to diminish relevant negative effects (26). Lack of psychological flexibility is closely related to the OCS in children and adults (3). ACT establishes psychological flexibility by six core processes: (1) acceptance is the willingness to accommodate and touch inner experience; (2) cognitive defusion makes behaviors not controlled by thoughts based on changing the relationship of thoughts, feelings, experiences, and self; (3) being present encourages people to fully accept the surrounding environments and own psychological activities without judgements; (4) self as context requires patients to pay attention to their real experiences by taking oneself as the context; (5) establish desired values; and (6) move toward the goal on the basis of values (27). Evidence shows the better effectiveness of ACT and the combination of ACT and drugs than drug alone treatment, whereas no significant difference in efficacy was observed between the ACT group and the combination treatment group (28). Shabani et al. (29) studied the differences between ACT or CBT combined drugs and drug alone therapy and found that the both combined groups can still significantly continue to improve symptoms after 8 weeks of treatment compared with drug alone. The patients in the ACT group showed improved psychological flexibility and were able to persist in mindfulness and maintain a valuable lifestyle after the treatment compared with those in the other two groups. In the short term, no significant difference in the improvement of OCS was observed between ACT and CBT. However, the former showed a more obvious improvement in the improvement of psychological flexibility.

Although rTMS and ACT are widely applied in the treatment of mental illness and can improve mental symptoms, not enough evidence exists to prove their definitive efficacy in OCD patients. In this study, OCD patients were randomly divided into rTMS and ACT groups, both of which took sertraline hydrochloride. Several scales, including Hamilton Depression Scale (HAMD), Hamilton Anxiety Scale (HAMA), Yale–Brown Obsessive Compulsive Scale (Y-BOCS), Symptom Checklist 90 (SCL-90), Acceptance and Action Questionnaire (AAQ-II), and Cognitive Fusion Questionnaire (CFQ), were used to evaluate the improvement of patients. This study is devoted to providing relevant evidence of the curative effect of OCD treatment and serves as a reference for the treatment direction.

## Materials and Methods

### Participants

Participants were recruited from inpatients at The First Affiliated Hospital of Nanchang University. From July 2019 to December 2019, 63 patients (aged 13–45 years old), who met diagnostic criteria of OCD in the fifth edition of DSM-5 and their scores of the Y-BOCS were at least 16, were randomly divided into the ACT group (*n* = 32) and rTMS group (*n* = 31). All patients had not received medication or psychotherapy before enrollment. During the experiment, in order to eliminate the interference of other drugs, all patients took sertraline only. The additional exclusion criteria were as follows: serious medical illnesses, other mental disorders, head trauma, seizures, electric convulsion therapy within 1 month, and visual or auditory disorder.

The study was approved by the Ethics Committee of The First Affiliated Hospital of Nanchang University. All participants signed the consent forms before the experiment.

### Methods

#### Drug Therapy

The selected inpatients with OCD were treated with sertraline hydrochloride (Zoloft). A small dose was provided, and it gradually increased to 200 mg/day according to the individual condition.

#### ACT Group

The group was treated with ACT by a professional psychotherapist twice a week for a total of eight sessions of 120 min.

#### rTMS Group

The group was treated with rTMS on the basis of drug treatment five times a week from Monday to Friday. All patients underwent baseline assessment of motor cortex excitability in both hemispheres by measuring the resting motor threshold (RMT). RMT was defined as minimum stimulus intensity when stimulating the motor cortex area, more than 5 times out of 10 times can make the corresponding target muscle (abductor pollicis brevis) produce a threshold EMG response (50 μV in peak-to-peak amplitude) (30). The stimulator device was a M-100 Ultimate TMS (YINGCHI, ShenZhen, China).

A meta-analysis compared the efficacy of different cortical targets in the treatment of OCD with rTMS, and demonstrated that LF-rTMS (1 Hz) applied over the SMA yields greater improvements in OCD severity than rTMS applied over the other areas (31). In this study, the parameters were 1 Hz frequency, 26-min sessions (four 5-min trains with an inter-train interval of 2 min, 1,160 pulses/d), at 100% of RMT. We used neuronavigation to locate the coil to the SMA. The coil and the handle were placed along the sagittal midline, 2 cm anterior to the reference, to stimulate the SMA bilaterally and simultaneously.

### Outcome Measures

The patients were evaluated by a professional psychiatrist. All scales were evaluated before treatment, 4 weeks posttreatment, and 8-week follow-up.

HAMD: This scale assesses the severity of depression of patients. HAMD consists of 24 symptom items: somatic symptom items (11) and mental symptom items (13).

HAMA: This scale is one of the most widely used test tools to assess the severity of anxiety in patients.

Y-BOCS: The scale's evaluation of the overall severity of patients has significant specificity, and clinical studies show good validity and reliability.

SCL-90: This scale consists of 90 items corresponding to nine factors evaluated by patients' own specific conditions in the week prior to assessments, involving thought, feeling, interpersonal relationship, and other aspects, and can systematically and comprehensively understand the spiritual outlook of patients.

AAQ-II: This scale assesses the degree of acceptance and experiential avoidance. The higher the total score is, the lower the degree of acceptance, and the higher the degree of experiential avoidance will be.

CFQ: This questionnaire was developed by Gillanders (32) in 2010 to assess the degree of cognitive fusion. The higher the total score is, the higher the degree of cognitive fusion will be.

The data was statistically analyzed by using SPSS software (version 26.0). The demographic characteristics for two groups adopted independent sample *t*-test and chi-square test. One-way ANOVA was used to compare the T-scores of SCL-90, Y-BOCS, HAMA, HAMD, AAQ–II, and CFQ for each group. For items with the homogeneity of variance, Fisher's Least Significant Difference in *post hoc* analysis was selected for processing, whereas Tamhane was selected for the post-analysis of items with the heterogeneity of variance. And sample *t*-test was used to compare the differences in various scales between two groups.

## Results

### Data Description of the Social Demographics

Before the experiment, two groups' age, gender, years of education and other variables were controlled to make the two groups comparable. The age and education years of the two groups were analyzed by independent sample *t*-test, and gender was analyzed by chi-square test. The results showed that there were no statistically significant differences between two groups on baseline demographic (*P* > 0.05) (Table 1).

**Table 1:** Demographic characteristics for two groups.

**Variable**	**ACT group (*N* = 32)**	**rTMS group (*N* = 31)**	**T/*X*^2^**	***P***
	**Mean (SD)**	**Mean (SD)**		
Age (years)	21.4 (8.4)	23.1 (9.6)	−0.761	0.449
Years of education	10.9 (2.7)	10.6 (2.4)	0.558	0.579
Gender (*n*, %)				
Male	19 (59.4)	19 (61.3)	0.024	0.877
Female	13 (40.6)	12 (38.7)		

### Data on the Clinical Symptoms and Psychological Flexibility Before Treatment

#### Comparison of Anxiety, Depression, and OCS Between the Two Groups of Patients With OCD

Sixty-three patients in two groups completed SCL-90, HAMA, HAMD, and Y-BOCS assessments before treatment. SCL-90 assessed the symptoms from 10 factors, such as somatization, obsessive–compulsive state, interpersonal relationship, depression, anxiety, hostility, phobia, paranoia, psychoticism, and other items, which initially reflected the severity of OCS, anxiety, and depression. No significant difference in the scores of the above-mentioned scales was observed between weeks 0 and 8 for the two groups (*P* > 0.05) (Tables 2, 3).

**Table 2:** Comparison of SCL-90 between the rTMS and the ACT groups.

**Item**	**rTMS group (*****n*** **=** **31)**		**ACT group (*****n*** **=** **32)**		***P*** **-value**	
**Factors**	**W0**	**W8**	**W0**	**W8**	**①**	**②**
	**Mean ± SD**	**Mean ± SD**	**Mean ± SD**	**Mean ± SD**		
Somatization	2.13 ± 0.72	1.59 ± 0.38	1.96 ± 0.76	1.46 ± 0.34	0.358	0.149
Obsessive state	3.22 ± 0.64	2.32 ± 0.32	2.98 ± 0.69	2.17 ± 0.42	0.159	0.117
Interpersonal relationship	2.88 ± 0.58	2.05 ± 0.41	2.62 ± 0.81	1.89 ± 0.45	0.152	0.153
Depression	3.14 ± 0.81	2.01 ± 0.42	2.80 ± 0.86	1.80 ± 0.40	0.114	0.047
Anxiety	3.05 ± 0.85	1.97 ± 0.44	2.89 ± 0.85	1.93 ± 0.44	0.477	0.695
Hostility	2.68 ± 0.70	1.91 ± 0.41	2.52 ± 0.88	1.73 ± 0.43	0.431	0.102
Terror	2.40 ± 0.90	1.63 ± 0.37	2.18 ± 0.57	1.61 ± 0.31	0.247	0.836
Paranoid	2.80 ± 0.74	1.85 ± 0.38	2.59 ± 0.74	1.81 ± 0.37	0.264	0.679
Psychotic	2.59 ± 0.61	1.85 ± 0.38	2.39 ± 0.71	1.67 ± 0.36	0.255	0.059
Other items	2.51 ± 0.57	1.92 ± 0.32	2.32 ± 0.64	1.60 ± 0.39	0.220	0.001

*W0 is before treatment; W8 means 8-week follow-up; ① indicates that the baseline WO values of the two groups are compared with each other; ② denotes that the W8 values of the two groups are compared with each other*.

**Table 3:** Comparison of anxiety, depression, OCS, and psychological flexibility in different groups.

	**rTMS group (*****n*** **=** **31)**		**ACT group (*****n*** **=** **32)**		***P*** **-value**	
	**WO**	**W8**	**W0**	**W8**	**①**	**②**
	**Mean ± SD**	**Mean ± SD**	**Mean ± SD**	**Mean ± SD**		
HAMA	24.19 ± 9.80	6.55 ± 4.07	23.13 ± 6.84	5.63 ± 3.48	0.617	0.337
HAMD	25.77 ± 9.20	6.97 ± 4.70	23.41 ± 6.33	5.38 ± 2.99	0.237	0.116
Y–BOCS	24.03 ± 7.68	15.00 ± 4.95	23.38 ± 8.33	17.75 ± 6.93	0.746	0.076
AAQ–II	37.23 ± 3.52	31.55 ± 3.00	37.50 ± 3.33	25.63 ± 3.13	0.752	0.000
CFQ	40.16 ± 5.56	33.39 ± 3.65	40.41 ± 5.29	26.78 ± 2.84	0.858	0.000

*W0 is before treatment; W8 is 8-week follow-up; ① indicates that the baseline WO values of the two groups are compared with each other; ② denotes that the W8 values of the two groups are compared with each other*.

#### Comparison of the Psychological Flexibility Between the Two Groups of Patients With OCD

The two groups evaluated the psychological flexibility in terms of acceptance and cognitive fusion. The patients in the two groups completed the AAQ-II and CFQ assessment tests, and the results showed no statistical significance (Table 3).

### Data on Clinical Symptoms and Psychological Flexibility After Treatment

#### Comparison of SCL-90 Evaluation in Each Stage Between the Two Treatment Groups

**Figure 1:**
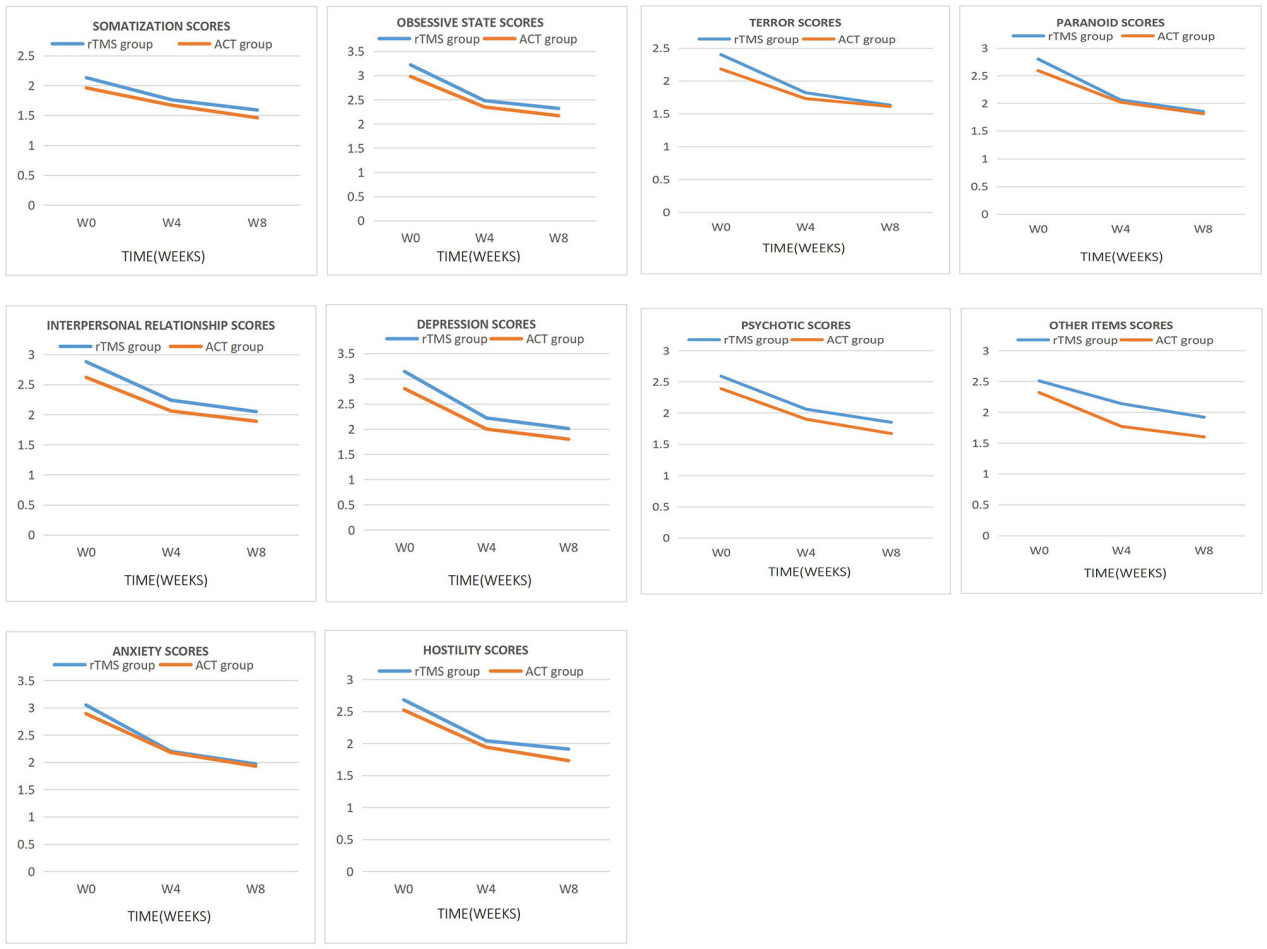
Main effect of time was observed for each factor scores of SCL-90.

The factors of SCL-90 assessment of two groups after 8 weeks of treatment were compared (Table 4; Figure 1). This study found a significant difference in depression factor scores and other items (*P* < 0.05); however, no significant difference in the remaining factor scores was observed between the two groups (*P* > 0.05). The SCL-90 scores of the patients in the two groups were compared after 8, 4 weeks, and before treatment (Table 4). The results showed no significant difference in the somatization factor scores between pretreatment and 4 weeks after treatment in the two group (*P* > 0.05); however, significant differences were observed in other factors for both groups (*P* < 0.05). A significant difference was observed in each factor score of SCL-90 in the two groups (*P* < 0.01) between pretreatment and 8 weeks after treatment, which pointed that both groups were gradually improved in anxiety, and their OCS were alleviated (Figure 1); however, no significant difference was observed between them after 4 and 8 weeks of treatment (*P* > 0.001).

**Table 4:** Comparison of SCL-90 symptom evaluation at different stages in the two group.

	**rTMS group (*****n*** **=** **31)**			**ACT group (*****n*** **=** **32)**			***P*** **value (rTMS group)**			***P*** **value (ACT group)**		
	**W0**	**W4**	**W8**	**W0**	**W4**	**W8**	①	②	③	①	②	③
	**Mean ± SD**	**Mean ± SD**	**Mean ± SD**	**Mean ± SD**	**Mean ± SD**	**Mean ± SD**						
Somatization	2.13 ± 0.72	1.76 ± 0.51	1.59 ± 0.38	1.96 ± 0.76	1.67 ± 0.44	1.46 ± 0.34	0.126	0.003	0.620	0.321	0.008	0.210
Obsessive state	3.22 ± 0.64	2.48 ± 0.46	2.32 ± 0.32	2.98 ± 0.69	2.35 ± 0.44	2.17 ± 0.42	0.000	0.000	0.551	0.000	0.000	0.469
Interpersonal relationship	2.88 ± 0.58	2.24 ± 0.50	2.05 ± 0.41	2.62 ± 0.81	2.06 ± 0.49	1.89 ± 0.45	0.002	0.000	0.116	0.010	0.000	0.640
Depression	3.14 ± 0.81	2.22 ± 0.49	2.01 ± 0.42	2.80 ± 0.86	2.00 ± 0.40	1.80 ± 0.40	0.000	0.000	0.396	0.000	0.000	0.259
Anxiety	3.05 ± 0.85	2.20 ± 0.50	1.97 ± 0.44	2.89 ± 0.85	2.18 ± 0.54	1.93 ± 0.44	0.000	0.000	0.303	0.001	0.000	0.257
Hostility	2.68 ± 0.70	2.04 ± 0.40	1.91 ± 0.41	2.52 ± 0.88	1.94 ± 0.44	1.73 ± 0.43	0.000	0.000	0.732	0.011	0.000	0.299
Terror	2.40 ± 0.90	1.82 ± 0.50	1.63 ± 0.37	2.18 ± 0.57	1.73 ± 0.26	1.61 ± 0.31	0.015	0.000	0.469	0.001	0.000	0.506
Paranoid	2.80 ± 0.74	2.06 ± 0.40	1.85 ± 0.38	2.59 ± 0.74	2.02 ± 0.39	1.81 ± 0.37	0.000	0.000	0.226	0.002	0.000	0.171
Psychotic	2.59 ± 0.61	2.06 ± 0.42	1.85 ± 0.38	2.39 ± 0.71	1.90 ± 0.38	1.67 ± 0.36	0.000	0.000	0.201	0.007	0.000	0.086
Other items	2.51 ± 0.57	2.14 ± 0.40	1.92 ± 0.32	2.32 ± 0.64	1.77 ± 0.45	1.60 ± 0.39	0.003	0.000	0.124	0.000	0.000	0.193

*① Is the comparison of WO and W4; ② is the comparison of W0 and W8; ③ is the comparison of W4 and W8; W0 is before treatment; W4 is 4 weeks posttreatment; W8 is 8-week follow-up*.

#### Comparison of HAMA, HAMD, and Y-BOCS Evaluations in the Two Groups After Each Stage

**Figure 2:**
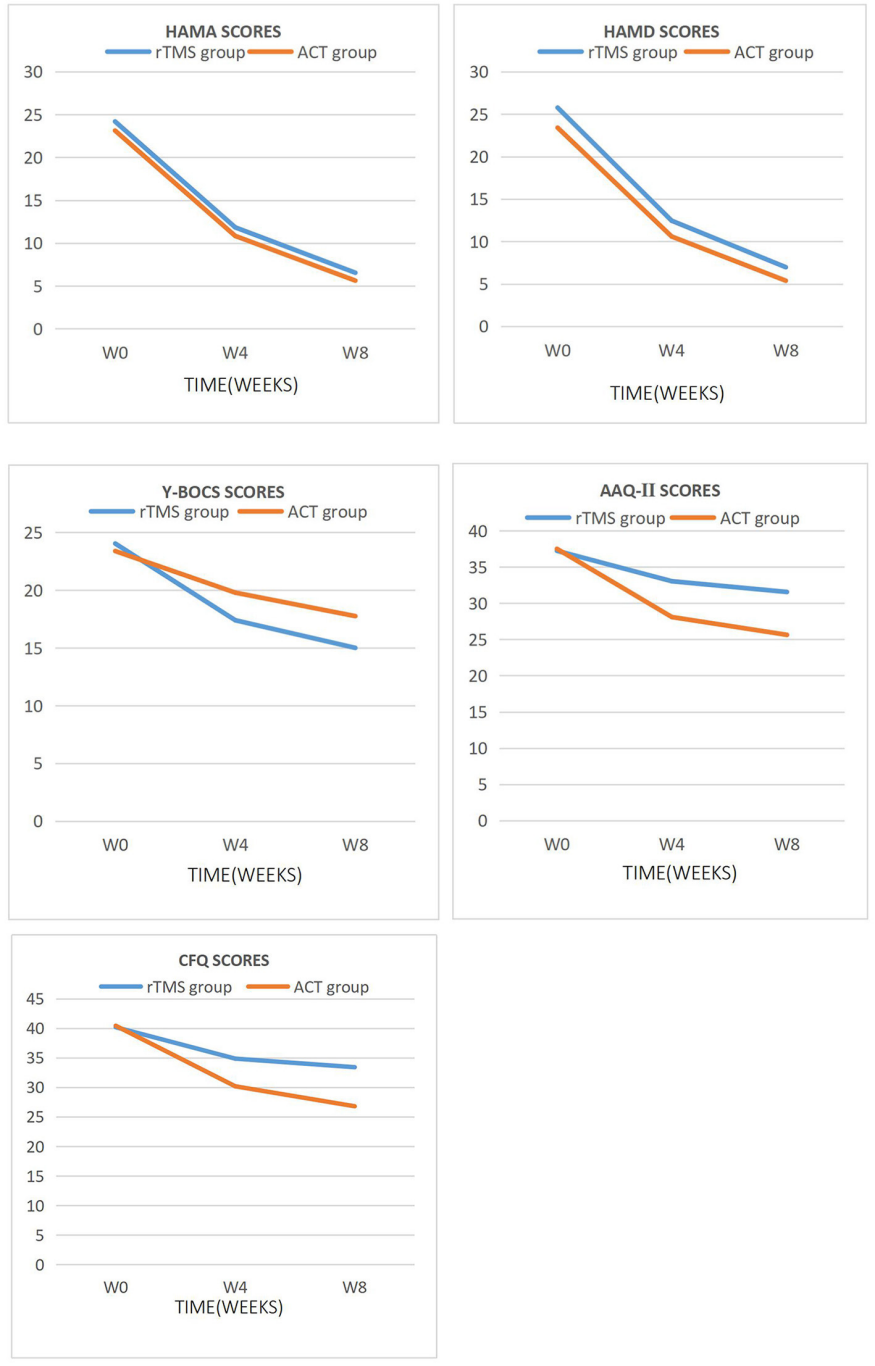
Main effect of time was observed for HAMA, HAMD, Y-BOCS, AAQ-II, and CFQ scores.

After treatment, the scores of HAMA, HAMD, and Y-BOCS in the two groups were compared (Table 5; Figure 2). The results showed no significant difference in the scores of the three scales mentioned above between the two groups (*P* > 0.05). The scores of HAMA, HAMD, and Y-BOCS of the two groups were compared before treatment and 4 weeks posttreatment. The results showed a significant difference in the scores of HAMA, HAMD, and Y-BOCS in the rTMS group (*P* < 0.01) and ACT group (*P* < 0.001). No significant difference was observed in the score of Y-BOCS (*P* > 0.05). Significant differences were observed in the scores of HAMA, HAMD, and Y-BOCS in the rTMS group (*P* < 0.001) and ACT group (*P* < 0.05) between pretreatment and 8 weeks after treatment. A significant difference was observed in the scores of HAMA and HAMD between the two groups after 4 weeks of treatment and 8 weeks of treatment; however, no significant difference was observed in the scores of Y-BOCS between the two groups (*P* > 0.05).

**Table 5:** Comparison of anxiety and depression, OCS, and psychological flexibility in the two group.

	**ACT group (*****n*** **=** **32)**			**rTMS group** ***n*** **=** **31)**			***P*** **-value (ACT group)**			***P*** **-value (rTMS group)**		
	**W0**	**W4**	**W8**	**W0**	**W4**	**W8**	①	②	③	①	②	③
	**Mean ± SD**	**Mean ± SD**	**Mean ± SD**	**Mean ± SD**	**Mean ± SD**	**Mean ± SD**						
HAMA	23.13 ± 6.84	10.84 ± 5.59	5.63 ± 3.48	24.19 ± 9.80	11.84 ± 6.45	6.55 ± 4.07	0.000	0.000	0.000	0.000	0.000	0.002
HAMD	23.41 ± 6.33	10.59 ± 4.70	5.38 ± 2.99	25.77 ± 9.20	12.45 ± 6.51	6.97 ± 4.70	0.000	0.000	0.000	0.000	0.000	0.003
BOCS	23.38 ± 8.33	19.78 ± 6.79	17.75 ± 6.93	24.03 ± 7.68	17.39 ± 5.55	15.00 ± 4.95	0.051	0.002	0.267	0.000	0.000	0.156
AAQ-II	37.50 ± 3.33	28.09 ± 3.18	25.63 ± 3.13	37.23 ± 3.52	33.03 ± 3.33	31.55 ± 3.00	0.000	0.000	0.003	0.000	0.000	0.077
CFQ	40.41 ± 5.29	30.16 ± 3.58	26.78 ± 2.84	40.16 ± 5.56	34.84 ± 4.24	33.39 ± 3.65	0.000	0.000	0.001	0.001	0.000	0.632

*① Is the comparison of WO and W4 in the two groups; ② is the comparison of W0 and W8; ③ is the comparison of W4 and W8; W0 is before treatment; W4 is 4 weeks posttreatment; W8 is 8-week follow-up*.

#### Comparison of AAQ-II and CFQ Evaluations in the Two Groups After Each Stage

After 8 weeks of treatment, the AAQ-II and CFQ scores were compared (Table 5; Figure 2), and significant differences were observed in the scores of the two scales between the two groups (*P* < 0.001). The AAQ-II and CFQ scores were compared before treatment, 4 weeks posttreatment, and 8-week follow-up. The results showed a significant difference in the AAQ-II and CFQ scores between the two groups before and 4 weeks after treatment (*P* < 0.001). A significant difference was observed in the AAQ-II and CFQ scores between the two groups before treatment and 8-week follow-up (*P* < 0.001). The scores of AAQ-II and CFQ in the ACT group were statistically significant (*P* < 0.01) compared with those in the 4- and 8-weeks of treatment. No significant difference was observed in the AAQ-II and CFQ scores in the rTMS group (*P* > 0.05).

## Discussion

Given that the pathogenesis of OCD is still obscured, the efficiency of the first-line treatment for OCD is still low. At present, the most effective treatment is cognitive behavioral therapy, that is, exposure and response prevention combined with high-dose SSRI drug therapy. However, studies have shown that such treatment is only effective for 40–60% of patients with OCD and does not achieve the effect of complete remission and cure (33). According to the study of application of rTMS in the refractory OCD patients by Pallanti et al. rTMS was effective in two-thirds of refractory patients, and this study demonstrated that rTMS can be used to treat OCD patients who are ineffective to SSRI drugs, and it may be more effective than antipsychotics (20). ACT is a representative treatment in the third wave of CBT, which is similar to ERP in many ways, and ACT enhances the efficacy of ERP by changing the way that patients interact with stimulus sources (34). A study of OCD and its pedigree disorders showed that the frequency of obsessive-compulsive symptoms occurrence was reduced and anxiety and depression were alleviated after eight ACT treatments (20). Our study have defined the ideas above. According to the Y-BOCS scores in the results of this study, the scores of the two groups decreased significantly in the 8-week follow-up (*P* < 0.05; Table 5; Figure 2), indicating that both rTMS and ACT can ease OCS. At the end of the fourth week of treatment, the Y-BOCS score of the rTMS group was significantly lower than that before treatment, which showed a statistical difference, and the ACT group also decreased, however, no statistical significance was observed (Table 5; Figure 2). The interventions of rTMS and ACT show improvement of symptoms, but the former takes effect faster.

According to the scores of AAQ-II and CFQ in the results of this study, the scores of the two groups decreased at the 4 weeks posttreatment and 8-week follow-up (Figure 2), indicating the improvement of psychological flexibility of patients with OCD intervened by rTMS and ACT. The scores of ACT group were compared at the 4 weeks posttreatment and 8-week follow-up of AAQ-II and CFQ. Statistical differences were observed; however, no statistical significance was observed in the rTMS group (Table 5). ACT taught patients to perform mindfulness breathing and other ways to actively and receptively deal with unnecessary thoughts or other psychological events, and therefore patients could adhere to training after treatment, while the rTMS group no longer continued related treatment 4 weeks later. ACT therapy can improve the psychological flexibility of patients for a long time compared with rTMS treatment. Some studies have shown that the lack of psychological flexibility is related to the severity of OCD symptoms (3). After 4 weeks of treatment, the prognosis of mental health is closely related to psychological flexibility (35, 36). Our study shows that ACT slowly improves symptoms in patients with OCD; however, they can show lasting improvement in the later stage as patients persist in training.

This study shows that the anxiety of patients of two groups has been improved after 4-week treatment judging from the SCL-90 and HAMA scores (*P* < 0.05; Tables 4, 5; Figures 1, 2). One meta-analysis of randomized controlled trials of rTMS in the treatment of OCD patients showed that active rTMS treatment significantly reduced the related anxiety symptoms of OCD patients (33). With the relief of obsessive-compulsive symptoms, anxiety was also improved, which is consistent with the results of foreign studies (35). ACT is able to accept phobic stimuli and live with them instead of avoiding them, so as to make room to pursue a valuable way of life and reduce anxiety, which is consistent with related studies (24). In addition, the depression of both groups also has improved according to the SCL-90 and HAMD scores (*P* < 0.05; Tables 4, 5; Figures 1, 2). Recently, a new type of TMS therapy has been used to improve depression. Low-frequency magnetic stimulation can regulate autonomic nervous dysfunction and metabolism without changing the action potential of neurons, which manifests great effects on modulating emotion (17). ACT guides patients to allow the existence of unwanted thoughts and immerse fully in what they are doing in the present rather than reflecting on the past and worrying about the future, and then patients' concomitant depression can be alleviated.

### Summary

The results of this study shows that the depression and anxiety of OCD patients can be improved after treatment. The two groups reveal that the treatment of OCD patients is effective, and the efficacy is equivalent. After the depression, anxiety and OCS were relieved and improved, the psychological flexibility of the two groups was also improved. However, the ACT group tried to explore a valuable way to live a full and meaningful life by making OCD patients hold an open and receptive attitude toward bad inner experiences rather than contradicting them. Therefore, the therapeutic effect of ACT on patients can be maintained and has a lasting effect on patients.

In summary, ACT and rTMS can help patients improve anxiety and depression and OCS as synergistic measures after giving drug treatment to OCD patients. ACT helps patients in dealing with unnecessary ideas and improving psychological flexibility through receptive and open attitude to improve patients' compliance with treatment.

### Limits

This study only analyzed the total score of Y-BOCS, but did not further analyze the scores of the two factor items (obsessions and compulsions) to explore the improvement of the two dimensions of patients with OCD and its mechanism. This study has no single SSRI treatment, ACT, and rTMS groups to compare them with the comprehensive treatment group for verifying the efficacy and advantages of comprehensive treatment. In addition, the follow-up time is short, and no long-term follow-up was conducted to trace the maintenance of late efficacy.

## Conclusion

Sertraline hydrochloride combined with ACT or rTMS can improve OCS, anxiety, depression, and psychological flexibility, and the effect is similar. However, ACT can last and effectively improve the psychological flexibility of patients with OCD.

## Data Availability Statement

The raw data supporting the conclusions of this article will be made available by the authors, without undue reservation.

## Ethics Statement

The studies involving human participants were reviewed and approved by the Ethics Committee of The First Affiliated Hospital of Nanchang University. Written informed consent to participate in this study was provided by the participants' legal guardian/next of kin.

## Author Contributions

JZ and SW devised the study concept and design, carried out data analysis, and made tables. XY, ZH, and JT recruited patients and collected data. JZ and SW prepared the manuscript which was revised by XY and ZH. MH supported funding, reviewed, and edited the paper. All authors contributed to the article and approved the submitted version.

## Funding

This study was financially supported by the National Natural Science Foundation of China (81960261).

## Conflict of Interest

The authors declare that the research was conducted in the absence of any commercial or financial relationships that could be construed as a potential conflict ofinterest.

## Publisher's Note

All claims expressed in this article are solely those of the authors and do not necessarily represent those of their affiliated organizations, or those of the publisher, the editors and the reviewers. Any product that may be evaluated in this article, or claim that may be made by its manufacturer, is not guaranteed or endorsed by the publisher.
